# Syncytial giant cell hepatitis associated with chronic lymphocytic leukemia: a case report

**DOI:** 10.1186/1471-2326-12-8

**Published:** 2012-07-19

**Authors:** Eva Gupta, Michael Yacoub, Martha Higgins, Ayad M Al-Katib

**Affiliations:** 1 Department of Medicine, St John Hospital and Medical Center, Detroit, MI, 48236, USA; 2 Department of Pathology, St John Hospital and Medical Center, Detroit, MI, 48236, USA; 3 Van Elslander Cancer Center, St John Hospital and Medical Center, 19229 Mack Avenue, Suite #36, Grosse Pointe Woods, MI, 48236, USA

## Abstract

**Background:**

Syncytial giant cell hepatitis (GCH) is an uncommon and an underreported disease entity. In two previously reported cases of GCH in patients with Chronic Lymphocytic Leukemia (CLL) liver failure ensued. Autoimmune and infective causes have been implicated but its etiology remains unclear.

**Case Presentation:**

A 60-year-old female with CLL presented with acute hepatitis with negative viral and auto-immune serologies and without any prior toxic exposure. Liver biopsy showed typical histological features of GCH. The patient was successfully treated with corticosteroids and intravenous immunoglobulin (IVIG). Her liver enzymes returned to baseline and have remained normal as of the last follow up almost 4 years later.

**Conclusions:**

Association of GCH with CLL may be under recognized. Clinical suspicion of GCH in CLL patients with serology-negative hepatitis, early liver biopsy and therapeutic intervention may influence outcome. This is the first case report of successful treatment of GCH in CLL patients. Moreover, our case also demonstrates the ability to resume effective CLL therapy post-GCH diagnosis without detriment to the liver.

## Background

Syncytial giant cell hepatitis (GCH) was first described by Phillips in 1991 [1]. This is an uncommon and often fulminant form of hepatitis characterized histologically by diffuse giant cell transformation of hepatocytes.

**Table 1:** Laboratory studies obtained at initial presentation

	**At presentation**	**Normal laboratory Reference values**
Hemoglobin (gm/dL)	12.3	12-16
WBC count (/uL)	237,800	3,500-10,000
Lymphocytes (%)	92	
Platelet Count (/uL)	233,000	150,000-350,000
Total Bilirubin (mg/dL)	4.2	0.3-1.2
Direct Bilirubin (mg/dL)	2.9	0-0.3
Alkaline Phosphatase (Units/L)	366	20-125
ALT (Units/L)	2070	0-35
AST (Units/L)	815	0-35
Total Protein (gm/dL)	5.4	6-7.8
Albumin (gm/dL)	4.1	3.5-5.5
Total Globulin (gm/dL)	1.3	2.5-3.5
LDH (Units/L)	547	40-100
INR	1.5	
Coombs’ Test	Negative	
Serum IgG (mg/dL)	493	751-1560
Serum IgA (mg/dL)	43	82-453
Serum IgM (mg/dL)	26	46-304

GCH is a rare condition found in 0.25% of liver biopsies in one study [2]. Giant cell transformation has been described in association with a number of diseases such as autoimmune hepatitis [3] with or without primary sclerosing cholangitis [4], *paramyxovirus* (measles) virus [1,5], herpes group of viruses [6], and hepatitis C [7]. In another series GCH was reported in 0.6% of liver biopsies done in patients with HIV [8,9]. There have been two separate case reports in the literature of GCH in patients with chronic lymphocytic leukemia (CLL). Fimmel et al. [5] reported GCH in a patient with CLL who progressed to cirrhosis. Alexpoulou [10] in 2003 reported a fatal case of GCH in a patient with CLL.

In this report, we describe a patient with CLL who developed GCH and was successfully treated with steroids and intravenous immunoglobulin (IVIG) along with her follow up for four years after the episode. This case suggests that early recognition, diagnosis and treatment of GCH in CLL patients may lead to favorable outcome.

## Case presentation

Our patient is a 60 year-old female with history of Rai stage II chronic lymphocytic leukemia (CLL) which was diagnosed in January 2004. She did well without treatment till August 2006. In August 2007, she presented with sudden onset of nausea, vomiting, abdominal pain, transaminitis and jaundice of 2-month duration. She denied any prior history of jaundice or blood transfusions. There was no history of travel. She had received 5 cycles of chemotherapy (oral chlorambucil plus prednisone) through January 2007. Rituximab was added due to progression of disease and continued till August 2007. Physical examination revealed mild icterus, generalized lymphadenopathy, right upper quadrant tenderness and splenomegaly without stigmata of chronic liver disease.

Peripheral blood smear revealed marked increase of small lymphocytes; many smudge cells and normocytic normochromic RBCs with mild anisocytosis. Serum acetaminophen and alcohol levels were negative. Serologies for hepatitis A, B, C, paramyxovirus, herpes virus, Epstein-Barr virus (EBV), and cytomegalovirus (CMV) were negative. Hepatitis C PCR was checked for confirmation and was found to be negative. Autoimmune workup for anti-nuclear antibody, anti-mitochondrial antibody, anti-smooth muscle antibody, anti-liver-kidney-microsomal antibody, and a coombs’ test was negative. Serum ferretin was 1196, iron 163 and percent saturation was 67.9. Serum copper was within normal limits. Computerized axial tomography scan of the abdomen revealed enlarged lymph nodes secondary to her known CLL but no biliary obstruction.

**Figure 1:**
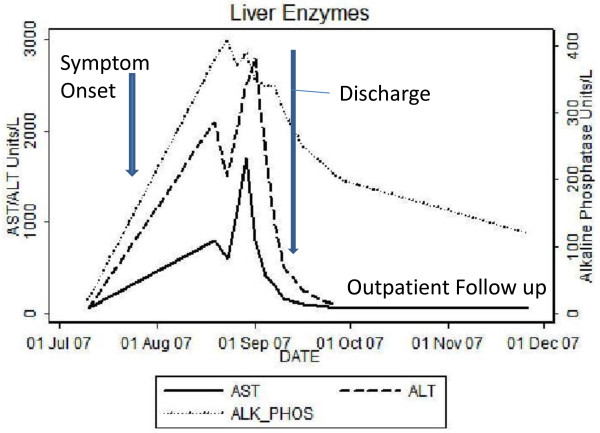
Liver Enzymes over disease course.

**Figure 2:**
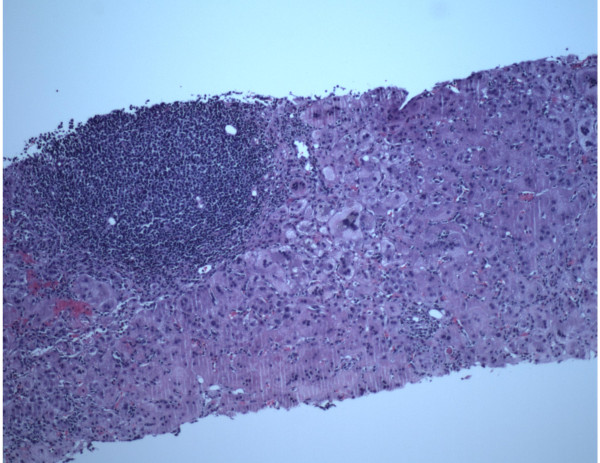
Giant cell hepatitis- light microscopy [10x].

**Figure 3:**
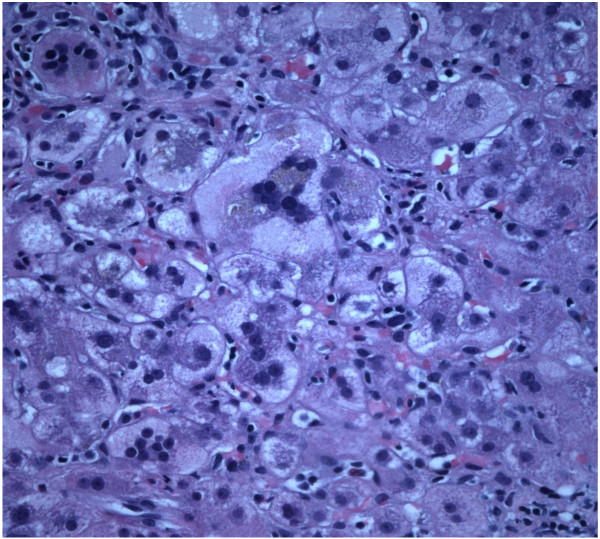
Giant cells on light microscopy [40x].

**Figure 4:**
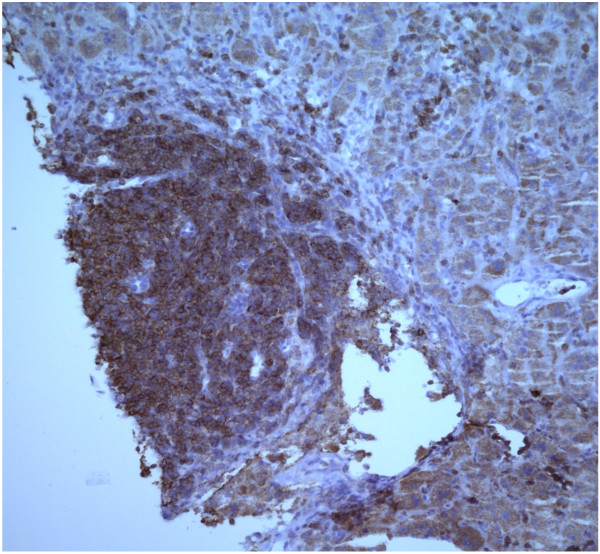
CD 5 immunostain.

**Figure 5:**
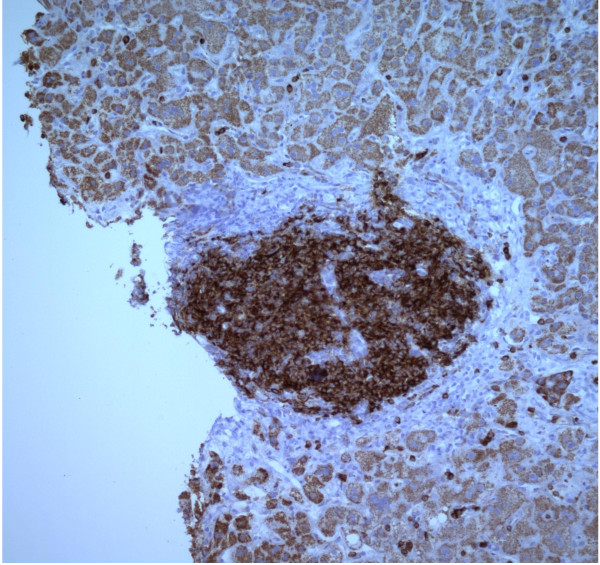
CD79a immunostain.

The patient’s alanine aminotransferase(ALT) peaked at 2776, aspartate aminotransferase(AST) peaked at 1471, alkaline phosphates at 419 within 9 days of presentation (Figure 1). INR peaked at 1.7. Liver biopsy was done which revealed syncytial giant cell hepatitis (GCH) with extensive periportal and subsinusoidal fibrosis along with infiltration by small lymphocytes (Figures 2, 3, 4 and 5).

The patient was treated with hydrocortisone 100 mg every eight hours for 9 days and intravenous immunoglobulin 1gm/kg body weight once every 4 weeks. She was discharged on oral prednisone 60 mg/day.

Post-discharge at day 36 after presentation her liver enzymes normalized (Figure 1). Patient’s CLL treatment was changed to a fludarabine (25 mg/m^2^ daily for 5 consecutive days), plus monthly rituximab (375 mg/m^2^ on day 1) and IVIG (1gm/kg BW) without signs of hepatitis. The patient developed warm antibody autoimmune hemolytic anemia (AIHA) in April 2008 with positive coombs test. Due to AIHA, fludarabine was replaced by oral cyclophosphamide 100 mg daily and monthly rituximab was continued. Her CLL therapy was changed in May 2011 to Bendamustine (90 mg/m^2^/day for 2 days in a row) with rituximab (375 mg/m^2^ IV on day 1). The patient has received 2 cycles as of July 2011 and has shown significant reduction in her splenomegaly, lymphadenopathy and her lymphocytosis. As of the last follow up in July 2011 her liver enzymes have remained normal. Treatment is fairly well tolerated although the patient has required G-CSF support.

## Discussion

GCH is a rare disorder that presents as acute hepatitis. It is characterized histologically by the presence of syncytial giant cells. Two major hypotheses proposing infective and autoimmune etiologies have been considered [6,11]. Its association with CLL was noted previously in two published reports. Fimmel et al. in 1998 published the first case of GCH in a patient with CLL [5]. There are significant differences between our case and that reported by Fimmel et al. CLL was concurrently diagnosed with GCH in Fimmel’s report in a patient with normal immunoglobulin levels. In contrast, our patient had an established diagnosis of CLL with hypogammaglobulinemia, and was on chemotherapy. In Fimmel’s case, the patient progressed to cirrhosis within one year of presentation. In our case early diagnosis and treatment has favorably influenced the course of liver disease. In the second case reported by Alexpoulou [10], a diagnosis of GCH was made only at post-mortem examination of the liver since the diagnosis was not suspected ante-mortem.

Similar to its etiology, the best treatment approach for GCH is also not clear. Variety of therapeutic interventions has been reported in the literature [6,8,11,12]. The most commonly used agents are corticosteroids with varying results. Pegylated interferon alpha-2b was used with ribavirin in a case of GCH associated with HIV infection with good results [8]. None of these therapeutic interventions, however, is consistently beneficial [9,13]. Patients with fulminant liver failure or late cirrhotic stages are usually treated with liver transplantation [6,11]. It is interesting however that disease was reported to recur in the transplanted liver [14].

GCH in patients with CLL presents special challenges to diagnose and treat. Detection of viral DNA by polymerase chain reaction (PCR) technique is highly recommended to definitively rule out viral etiologies. Also the immune compromised condition of CLL patients whether disease- or treatment-related can make viral serology studies unreliable. After excluding known causes of acute hepatitis, GCH should be suspected. GCH is a pathological diagnosis and liver biopsy should be performed. Electron microscopy should be routinely included in the planning of liver biopsy since biopsy material may not be adequate for subsequent testing.

In deciding on how to best treat GCH in CLL patients, several factors related to the underlying CLL should be taken into consideration. In our case, the finding of hypogammaglobulinemia made IVIG an obvious choice. CLL is a disease known to be associated with hypogammaglobulinemia in up to 30% of the cases and where IVIG is beneficial as adjunct in the management of infectious complications [15,16]. Our patient was also treated with steroids, which have been commonly used in GCH [13] and are useful in CLL [17]. With the development of Coomb’s-positive autoimmune hemolytic anemia (AIHA), treatment was switched to oral cyclophosphamide and rituximab to avoid exacerbation of AIHA by fludarabine. Evidence supporting the use of cyclophosphamide and rituximab is present in the pediatric age group for the treatment of GCH [12] and the same regimen was used successfully in the treatment of fludarabine-associated AIHA in CLL [18].

## Conclusions

In conclusion, our case brings to attention the association between CLL and GCH. High index of suspicion is required in serology-negative acute hepatitis in patients with CLL. In contrast to similar cases reported by Fimmel and Alexpoulous where the disease either progressed to cirrhosis or fulminant hepatic failure, our patient responded well to therapy and was able to resume treatment for CLL. While there is no evidence to date demonstrating increased risk of CLL patients to GCH, the host environment in CLL is conducive of GCH, i.e. association with auto antibodies and susceptibility to infections. Recognizing such cases is crucial to the development of suitable therapeutic strategies.

## Consent

Informed consent was obtained from the patient for publication of this case report and any accompanying images.

## Competing interests

The authors of this manuscript declare that they have no competing interests.

## Authors’ contributions

EG, MY and AA were responsible for assessment and management of the patient. MH was responsible for pathology review. EG and AA reviewed the literature. AA and EG wrote the manuscript. All authors contributed towards the preparation of this manuscript. All authors read and approved the final manuscript.

## Pre-publication history

The pre-publication history for this paper can be accessed here:

http://www.biomedcentral.com/1471-2326/12/8/prepub
